# Supplementary material for: Outer membrane translocation of pyocins via the copper regulated TonB-dependent transporter CrtA
Source: Biochem J. 2023 Jul 17;480(14):1035–49. doi: 10.1042/BCJ20220552 (PMC10422930; doi:10.1042/BCJ20220552)
Supplement: Supplementary Material [file BCJ-480-1035-s1.pdf]

Supplementary Information

Table S1. Genomic coordinates of P-box element, pyocin gene and immunity protein gene for the pyocin SX1 and SX2 operons.

Pyocin	Assembly accession	P-box position	Pyocin gene position	Immunity gene position
SX1-ImSX1	GCF_003837245.1	23085 – 23132 (- strand)	23204 – 25256 (- strand)	25261 – 25516 (- strand)
SX2-ImSX2	GCF_000233495.1	8641 – 8688 (+ strand)	8761 – 10852 (+ strand)	10852 – 11113 (+ strand)

Table S2. Pyocin sensitivity and affected genes in spontaneous pyocins SX1/SX2 resistant mutants

Isolate	Sensitivity to pyocin				Affected gene	Gene product	Mutation type*
	SX1	SX2	SD2	L1			
Wild type	S	S	S	S	ND	ND	ND
SX1-R17	R	R	S	S	*pa0434*	Putative TonB-dependent transporter	1036 G del (nonsense)
SX1-R20	R	R	S	S	*pa0434*	Putative TonB-dependent transporter	68 G in (nonsense)
SX2-R5	R	R	S	S	*pa0434*	Putative TonB-dependent transporter	898 C→T (nonsense)
SX2-R11	R	R	S	S	*pa0434*	Putative TonB-dependent transporter	1764 C del (nonsense)
SX2-R1	T	R	T	R	*gmd*	GDP-mannose 4,6-dehydratase	450 C→G (nonsense)
SX2-R14	T	R	T	R	*gmd*	GDP-mannose 4,6-dehydratase	379 A→T (missense)
SX1-R6	T	R	T	R	*gmd*	GDP-mannose 4,6-dehydratase	+316 G del (regulation site)
SX2-R3	T	R	T	T	*pa5455*	Putative glycosyltransferase	866 T del 868 C→A (nonsense)

The pyocin sensitivity was determined by size of clear zone produced by spot plate assay. All pyocins were tested at the concentration of 1 mg ml^{-1}. S = sensitive; R = resistant and T = tolerant. ND indicates not detected. * The number indicates the nucleotide position of the gene, + indicates upstream position from the gene; del = deletion; in = insertion; → = base substitution.

Table S3 Strains used in this study

Strain	Relevant characteristics	Reference or source
E. coli		
DH5α	*F-, φ80dlacZΔM15, Δ (lacZYAargF) U169, deoR, recA1, endA1, hsdR17(rk-, mk+), phoA, supE44, λ thi-1, gyrA96, relA1*	ThermoFisher Scientific
BL21(DE3)pLysS	F- *ompT hsdS*B (rB-mB-) *gal dcm* (DE3) pLysS (CamR)	Agilent Technologies
P. aeruginosa		
PAO1	Clinical isolate, burn wound	(1)
PAO1Δ*PA0434*	Transposon insertion mutant	(1)
PAO1Δ*tonB1*	Transposon insertion mutant	(1)
PAO1Δ*tonB2*	Transposon insertion mutant	(1)
PAO1Δ*tonB3*	Transposon insertion mutant	(1)
PAO1Δ*ftsH*	Transposon insertion mutant	(1)
P7	Clinical isolate, paediatric CF patient	(2)

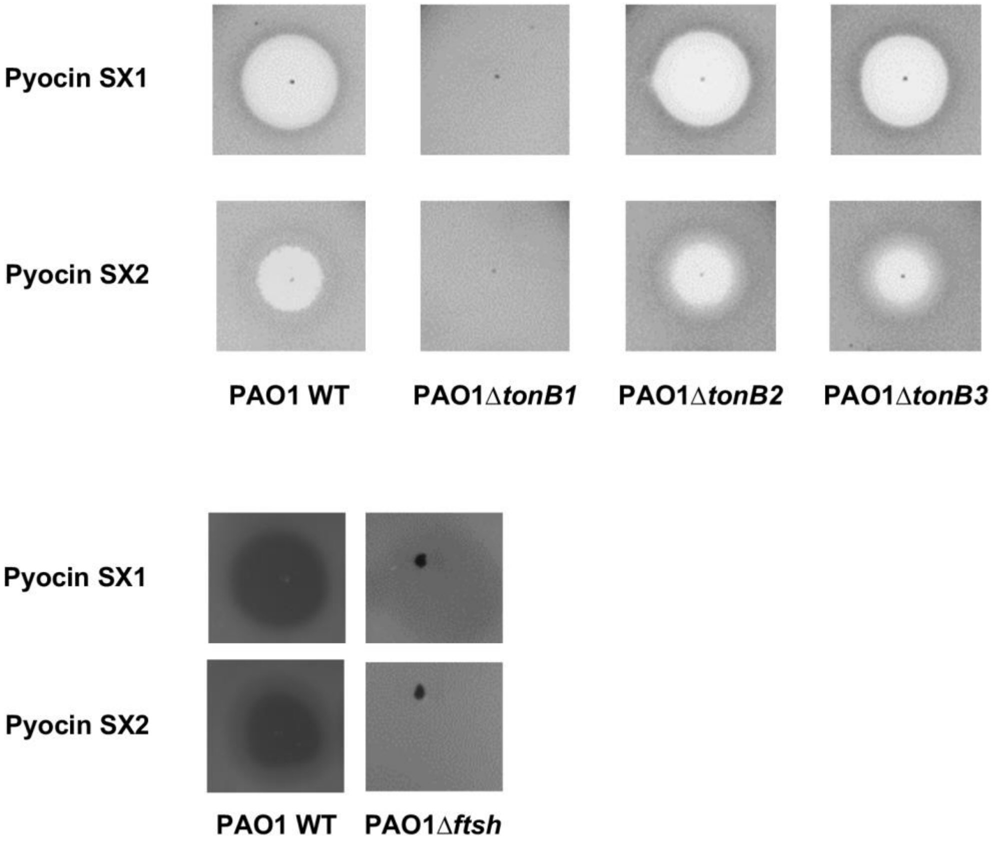

Figure S1 The killing activity of pyocins SX1/SX2 depend on TonB1 and FtsH. All pyocins were tested at the concentration of 1 mg ml^{-1}.

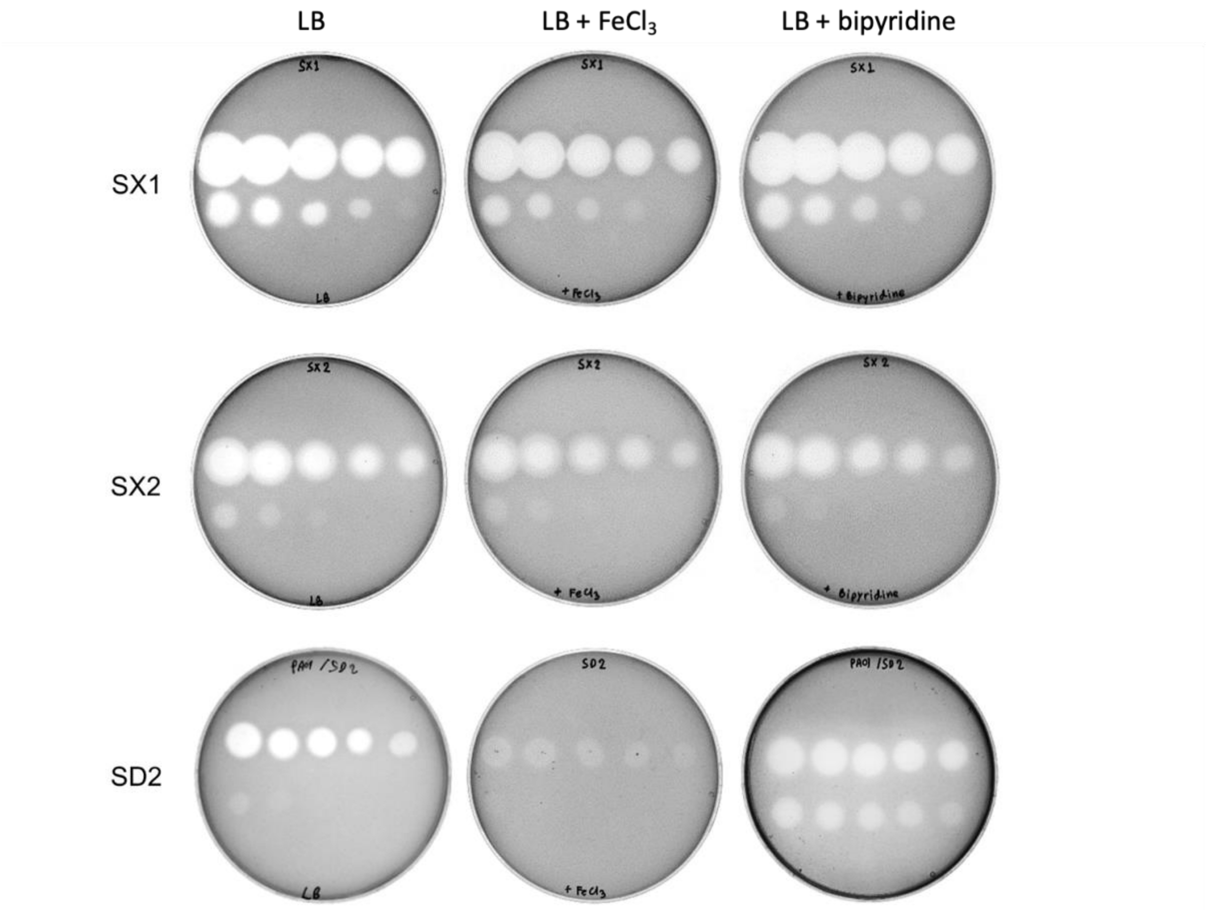

Figure S2. Overlay spot plate assay of pyocins SX1/SX2 and SD2 under iron-rich and iron-limited conditions. The pyocins were serially diluted from 1000 to 0.051 µg ml^{-1} (3X dilution) and 5 µl were spotted on the LB agar plates. For iron rich media 50 µM $FeCl_3$ was added to the LB agar and for iron limited conditions 200 µM bipyridine was added.

References

1. Held K, Ramage E, Jacobs M, Gallagher L, Manoil C. Sequence-verified two-allele transposon mutant library for Pseudomonas aeruginosa PAO1. J Bacteriol. 2012;194(23).
2. McCaughey LC, Ritchie ND, Douce GR, Evans TJ, Walker D. Efficacy of species-specific protein antibiotics in a murine model of acute Pseudomonas aeruginosa lung infection. Sci Rep. 2016;6.
